# Potential envenomation by the aglyphous pseudoxyrhophiine snake *Leioheterodon madagascariensis* and description of its dentition

**DOI:** 10.1186/s40409-015-0047-2

**Published:** 2015-11-20

**Authors:** Bertrand Razafimahatratra, Cynthia Wang, Akira Mori, Frank Glaw

**Affiliations:** Département de Biologie Animale, Université d’Antananarivo, BP 906, Antananarivo, 101 Madagascar; Zoologische Statssammlung München (ZSM-SNSB), Münchhausenstrabe 21, 81247 München, Germany; Department of Zoology, Graduate School of Science, Kyoto University, Sakyo, Kyoto, 606-8502 Japan

**Keywords:** Madagascar, Lamprophiidae, Pseudoxyrhophiinae, *Leioheterodon madagascariensis*, Envenomation, Dentition, Microcomputed tomography

## Abstract

We report on a case of potential envenomation caused by multiple bites by the aglyphous opisthodont snake *Leioheterodon madagascariensis* in the left thumb of a healthy adult man, which is among the most serious snakebites hitherto reported from Madagascar. The adult snake (total length > 1 meter) was unusually aggressive before and during capture. The symptoms included extensive bleeding, severe local pain, and substantial swelling of the hand and the distal part of the lower arm. The swelling disappeared entirely after five days, but pain in the thumb (when moved) was recognizable even longer. Although *L. madagascariensis* is widespread and common in anthropogenic habitats in eastern and western Madagascar, this case report seems to be the first description of long-lasting symptoms of its bite. Since aglyphous snakes are relatively rarely involved in “envenomation” and because hemolytic activity has been recorded in the secretions of the Duvernoy’s glands of *Leioheterodon,* we describe its dentition using microcomputed tomography and discuss the potential mode of envenomation in this case.

**Electronic supplementary material:**

The online version of this article (doi:10.1186/s40409-015-0047-2) contains supplementary material, which is available to authorized users.

## Background

The caenophidian snake fauna of Madagascar consists mainly of a large radiation of lamprophiid (mostly pseudoxyrhophiine) snakes currently comprising more than 80 endemic species in 20 genera, and new species continue to be discovered and described regularly [1, 2]. Although relatively closely related to highly venomous snakes of the family Elapidae, which includes cobras, kraits, and sea snakes, lamprophiid snakes from Madagascar are comparatively harmless, and none of these snakes has venomous front fangs [3]. Several Malagasy genera are aglyphous, whereas others are opisthoglyphous, i.e., they have grooved rear fangs and are considered as mildly venomous, but little is known on the effects of their venoms to humans [4, 5]. Until now, mild “envenomation” caused by accidental bites by Malagasy snakes has been reported only for the psammophiine species *Mimophis mahfalensis* [6, 7] and six pseudoxyrhophiine species: two species of *Madagascarophis* [6, 8], *Leioheterodon modestus* [5], *Leioheterodon madagascariensis* [9], *Ithycyphus miniatus* [10], and *Langaha madagascariensis* [11], all of which can attain a relatively large size (≥1 m total length).

*Leioheterodon* comprises three robust species with terrestrial and diurnal habits [12, 13]. Although it is an aglyphous genus, it has a pair of enlarged teeth on the posterior end of the maxilla [4–6]. *Leioheterodon madagascariensis*, the Malagasy giant hognose snake, is one of the largest (total length up to > 1.50 m) and most common snakes in natural and anthropogenic habitats in eastern and western Madagascar, and is well known throughout the island as “menarana” [13, 14]. It is endemic to Madagascar and has been introduced to the island of Grand Comoro [15–17]. The recorded diet includes fish, frogs, reptiles (including snakes), reptile eggs, mammals and birds [14, 18–20]. According to Conant [21] one female of *L. madagascariensis* in captivity constricted larger prey and swallowed small animals directly without constriction. Mori and Tanaka [22] observed prey handling behavior of juvenile *L. madagascariensis* and reported that virtually all prey animals were swallowed alive. These observations suggest that the biological function of the secretions of the Duvernoy’s glands is not to kill prey during feeding. On the other hand, local pain, and mild swelling caused by the accidental bite by *L. madagascariensis* on humans is reported [9].

Herein, we report on a case of snakebite by *L. madagascariensis* in an adult man with a detailed description of the symptoms that were localized to the bite wound on the hand and the distal part of the arm. No other complications were observed. The potential mode of envenomation by Duvernoy’s gland secretions is discussed with reference to a study of its aglyphous dentition based on microcomputed tomography.

## Case presentation

During intensive ecological fieldwork on snakes in Madagascar, the first author (hereafter referred to as patient) has been bitten several times by *L. madagascariensis* in the past decade. In all these cases only minor bleeding was observed after the bite, and only a small area around the puncture wounds became swollen. Only little pain was felt, and all symptoms had disappeared after about one hour.

**Fig. 1:**
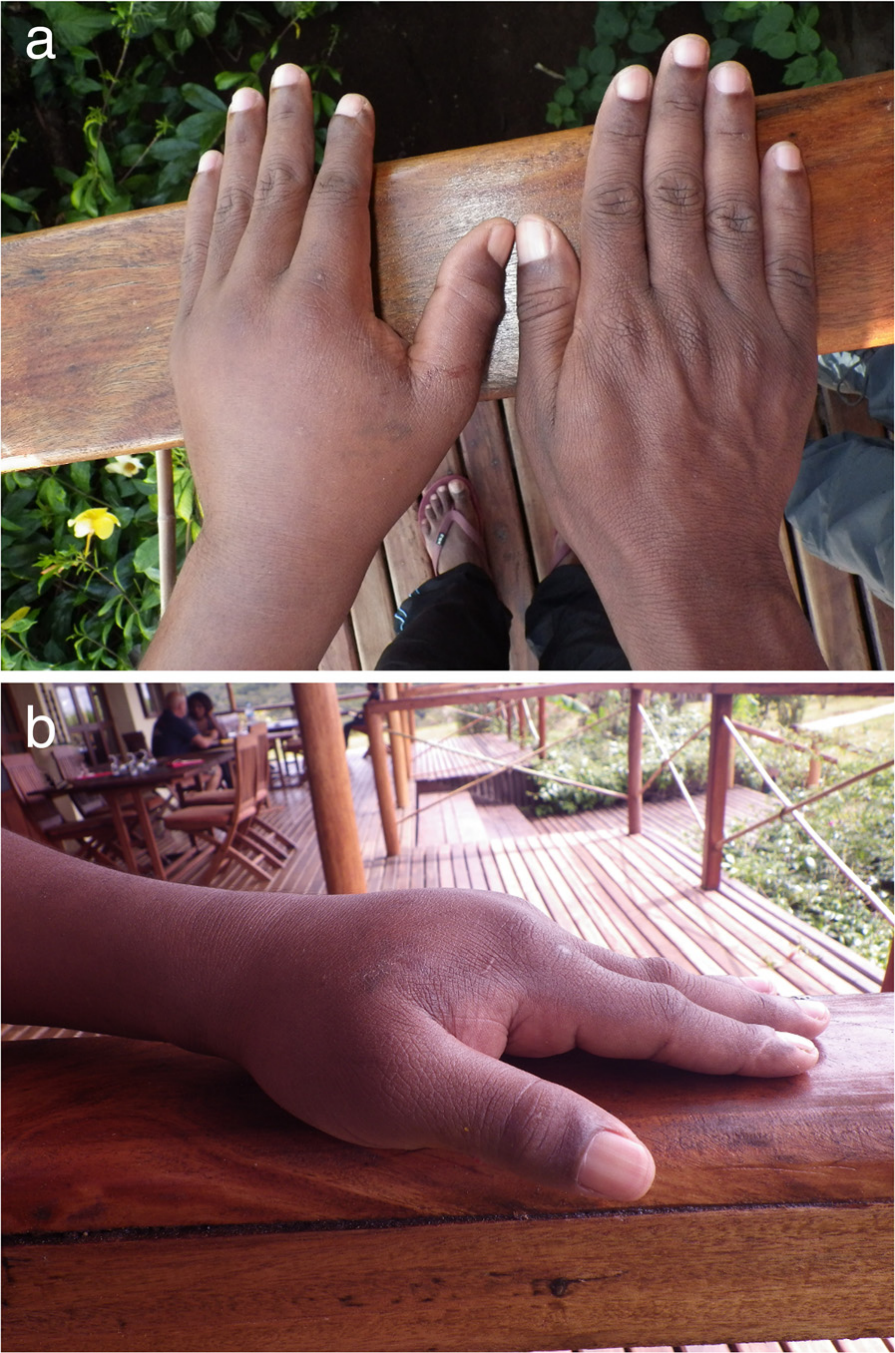
Photographs showing the swollen left hand after the bite of *Leioheterodon madagascariensis*. Approximately (**a**) 5 h and 30 min and (**b**) 24 h after the accident

On 10 May 2014, during fieldwork in northern Madagascar, the patient (41 years old, 58 kg) captured an adult *L. madagascariensis* (total length > 1 m) at 11:40 a.m. While his left hand was wrapped around the snake’s neck, the snake bit dorsolaterally (on the right side) the middle segment of the left thumb three times with increasing severity. Total bite duration was approximately 90 s. Shortly after the removal of the snake, the first series of photographs were taken to document the symptoms (Fig. 1). After removing the blood and disinfecting the thumb with an alcoholic solution (Octenisept®), three puncture wounds became visible. Two of them appeared rather deep with a distance of about 2 mm from each other, whereas the third one was relatively shallower with a distance of about 4 mm to the next one.

Initial symptoms included partial loss of feeling in the fingers and distal half of the arm. Bleeding lasted for more than three hours after the initial bite. Swelling extended throughout the hand and to the distal half of the arm, and reached its maximum extent in 24 h. Slight pain was recognized in the left axillary lymph nodes five hours after the bite and persisted until the third day. Swelling lasted five days, but the last symptoms, the pain in the thumb and in the hand, only completely disappeared by the evening of 19 May 2014, nine days after the bite. During the whole event, only local symptoms were recognized. There were no signs of fever, diarrhea, headaches, sweating, bad sleep, or lethargy, and appetite remained normal. Treatment only consisted of cleaning up of the blood, disinfecting the wound, and application of a brayed local medicinal plant (called “tamotamo” in Malagasy language) to the injured region of the thumb eight hours after the first bite.

### Dentition

The head of a *L. madagascariensis* was subjected to microtomographic analysis at the Zoologische Staatssammlung München (ZSM) using a phoenix nanotom m (GE Sensing & Inspection Technologies, Billerica, MA, USA) at 130 kV and 100 μA for 18 min, generating 1440 projections per scan. The data were visualized in VG Studio Max 2.2 (Visual Graphics GmbH, Heidelberg, Germany). Surface meshes of the skull were generated using the threshold tool in the segmentation editor of the software AMIRA 5.4.5 (FEI Visualization Sciences Group, Burlington MA, USA). The PDF-3D model (see Additional file 1) was then prepared following the procedures outlined by Ruthensteiner and Hess [23].

**Fig. 2:**
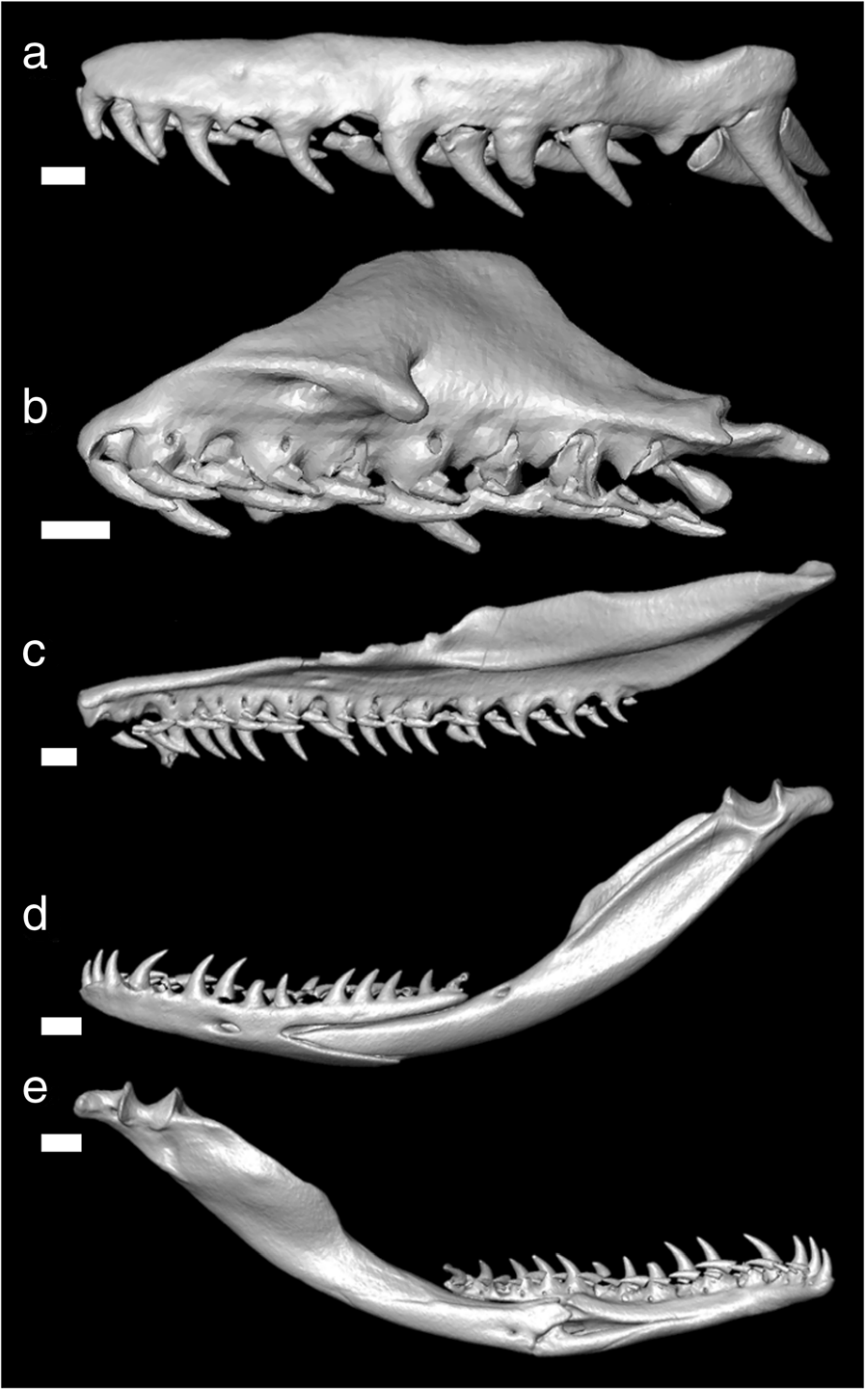
Dentition of *Leioheterodon madagascariensis* (ZSM 806/2001) based on microcomputed tomography scans of the skull. Outer lateral views of: (**a**) maxilla, (**b**) palatine, (**c**) pterygoid, (**d**) mandible (outer lateral view); (**e**) inner lateral view of mandible. Scale bars = 1 mm for (**a**–**c**). Scale bars = 2 mm for (**d**) and (**e**)

The following dentitional description is based on one adult male (ZSM 806/2001), of which separated dentigerous bones are shown in Fig. 2. On the maxilla, the 12 teeth increase in size posteriorly and are separated from the paired rear teeth by a diastema. Maxillary diastema width is more than the width of the preceding tooth. The paired rear teeth are greatly enlarged, approximately 4 mm in length (1.6 times the size of the preceding tooth), and are ungrooved with a knifelike posterior edge for a majority of teeth. The tips of the leading edge are slightly compressed. The nine palatine teeth are subequal. The 22 pterygoid teeth decrease in size. The dentary has 17 teeth, with the anterior dentary teeth slightly larger than the posterior.

An interactive, 3D reconstructed skull of another *L. madagascariensis* (ZSM 806/2001) can be found in Additional file 1.

## Discussion and conclusions

The case of snakebite by *L. madagascariensis* described herein is among the most serious hitherto reported from Madagascar. The swelling lasted for more than four days, and the last symptoms (local pain in the thumb) disappeared only after nine days. The comparatively strong swelling and heavy pain may be partially attributed to the amount of blunt trauma experienced by the patient (three bites total, with two deep bites). Additionally, the patient has a prior history of snakebites during which he could have developed a hypersensitivity reaction to the gland secretions, which then in combination with the prolonged duration of the bite, could have further contributed to the relative severity of the case. The snake was rather aggressive prior to capture, and although speculative, an increasing stress level may have resulted in more secretion delivery in the moment of biting.

It is likely that some of the symptoms could be attributed to secretions of the Duvernoy’s glands. Although no description of these glands in *L. madagascariensis* is available, it was recently described in *Mimophis mahfalensis*, a species of psammophiine lamprophiid snake [7]. Hemolytic activity has been recorded when the Duvernoy’s gland secretion from *L. geayi* was mixed with blood from mice, rabbit, and chicken, and injections of gland secretion from *L. geayi* into mice resulted in paralysis and, ultimately, death within twenty minutes [24]. Thus, Domergue [6] considered *Leioheterodon* to be capable of “envenomation” [5]. However, it appears unlikely that the “venom” of the “menarana” is mainly used for defense of potential predators. Instead it might be used primarily to aid in feeding by incapacitating large prey before it is swallowed or by facilitating digestion [25]. Further study is required to evaluate the composition of Duvernoy’s gland secretions and their toxicity to humans.

The dentition of *L. madagascariensis* appears to be relatively generalized and lacks specialized teeth for specific prey types found in previous studies [26–29]. This is supported by its generalist diet of prey ranging from frogs to birds to iguana eggs [14]. The left maxilla of *L. modestus* has been figured by Mori [5], which resembles that of *L. madagascariensis*. Examinations of the “venom” delivery mechanism of an opisthoglyphous snake, *Boiga irregularis*, which possesses Duvernoy’s glands and grooved, enlarged maxillary teeth, clarified the low-pressure system and found it to be much less efficient in venom delivery than the high-pressure system of viperids and elapids [30, 31]. The mechanism of “venom” delivery in snakes with ungrooved, enlarged teeth is not clear, but is probably similar to the low-pressure system of opisthoglyphous snakes in which the saliva flows into the bite wound. Further investigations of the mechanism of envenomation may reveal the functional role of ungrooved but enlarged maxillary teeth of *Leioheterodon*.

## Consent

Written informed consent was obtained from the patient for publication of this case report and accompanying images. A copy of the written consent is available for review by the editor-in-chief of this journal.

## Ethics committee approval

The CT-scanned snake specimen was collected and exported with all necessary permits of the Ministère de l’Environnement, de l’Ecologie, de la Mer et des Forêts, Madagascar. Approval by an ethics committee is not required by Malagasy laws for observations of accidental snakebites.

## Additional file

Additional file 1:
**Interactive PDF-3D model based on the computed tomography scanned skull of** ***Leioheterodon madagascariensis*** **(ZSM 806/2001).** Click on the image to enable the 3D content using Adobe Acrobat Reader (version IX or later). (PDF 1376 kb)
