# Supplementary material for: Potential envenomation by the aglyphous pseudoxyrhophiine snake Leioheterodon madagascariensis and description of its dentition
Source: J Venom Anim Toxins Incl Trop Dis. 2015 Nov 20;21:47. doi: 10.1186/s40409-015-0047-2 (PMC4654879; doi:10.1186/s40409-015-0047-2)

Supplementary Figure S1

Interactive PDF 3D model of the skull of the CT scanned *Leioheterodon madagascariensis* (ZSM 806/2001). Click image in Adobe Acrobat or Reader (version IX or later) to enable the 3D contents.

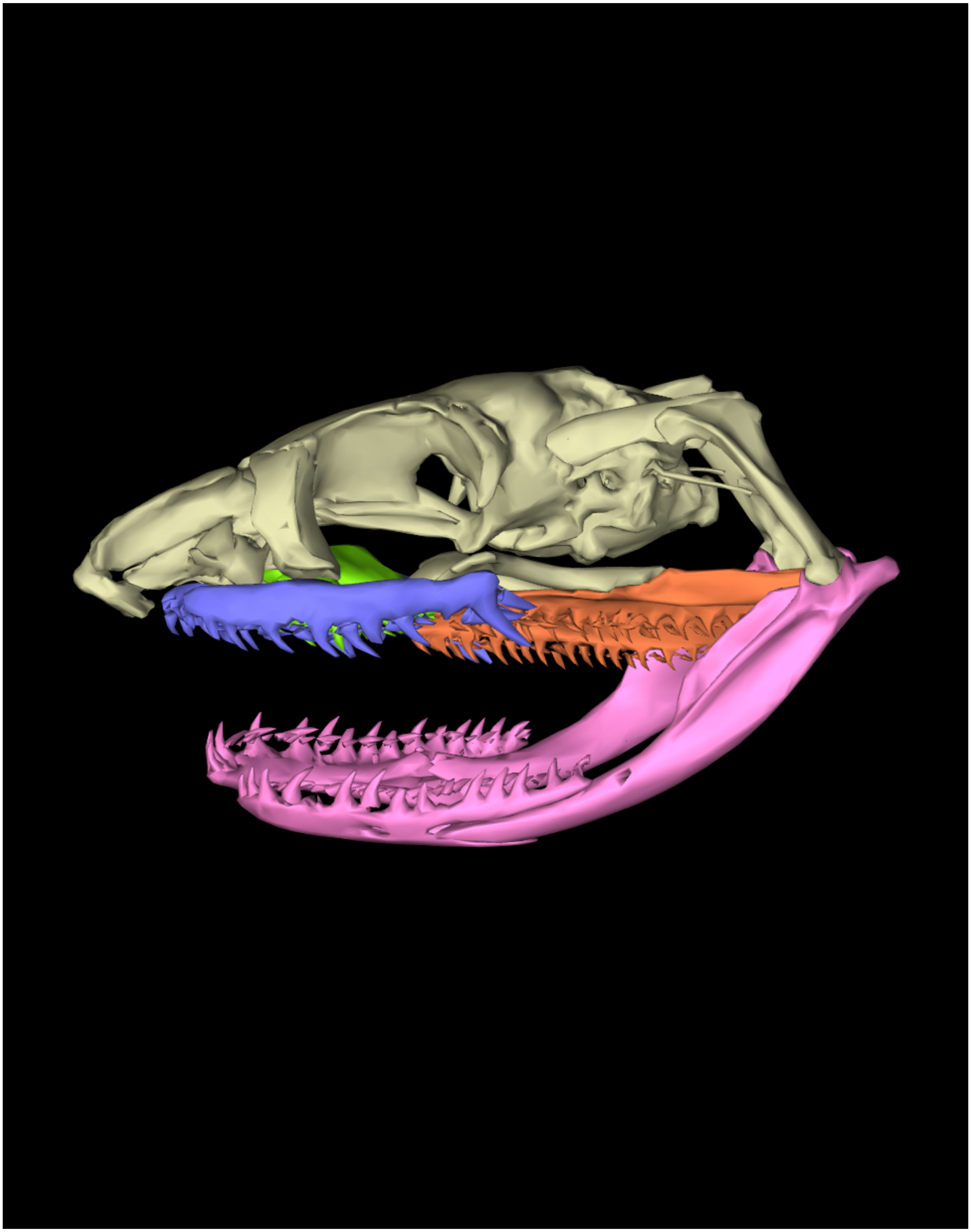

Supplement: Additional file 1: — Interactive PDF-3D model based on the computed tomography scanned skull of Leioheterodon madagascariensis (ZSM 806/2001). Click on the image to enable the 3D content using Adobe Acrobat Reader (version IX or later). (PDF 1376 kb) [file 40409_2015_47_MOESM1_ESM.pdf]
